# Myocardial transcriptomic analysis of diabetic patients with aortic stenosis: key role for mitochondrial calcium signaling

**DOI:** 10.1186/s12933-024-02329-5

**Published:** 2024-07-08

**Authors:** Maelle Cherpaz, Emmanuelle Meugnier, Gaultier Seillier, Matteo Pozzi, Romain Pierrard, Simon Leboube, Fadi Farhat, Marco Vola, Jean-François Obadia, Camille Amaz, Lara Chalabreysse, Chloe May, Stephanie Chanon, Camille Brun, Lucas Givre, Gabriel Bidaux, Nathan Mewton, Genevieve Derumeaux, Cyrille Bergerot, Melanie Paillard, Helene Thibault

**Affiliations:** 1 Explorations Fonctionnelles Cardiovasculaires, Hospices Civils de Lyon, 69500 Bron, France; 2 Laboratoire CarMeN - IRIS Team, INSERM, INRA, Université Claude Bernard Lyon-1, Univ-Lyon, 69500 Bron, France; 3 Service de Cardiologie, CHU Nord, 42100 Saint-Étienne, France; 4 Laboratoire d’anatomopathologie, Hospices Civils de Lyon, 69500 Bron, France; 5 Chirurgie Cardiaque, Hospices Civils de Lyon, 69500 Bron, France; 6 Centre d’investigation Clinique, Hospices Civils de Lyon, 69500 Bron, France; 7 Present Address: INSERM U955, Université Paris-Est Créteil, Créteil, France; 8 Present Address: Department of Physiology, AP-HP, Henri Mondor Hospital, FHU SENEC, Créteil, France

**Keywords:** Pressure overload, Mitochondria, RNAseq, MAM, Mitochondria-associated membranes, MICU1, MCU, HFpEF

## Abstract

**Background:**

Type 2 diabetes (T2D) is a frequent comorbidity encountered in patients with severe aortic stenosis (AS), leading to an adverse left ventricular (LV) remodeling and dysfunction. Metabolic alterations have been suggested as contributors of the deleterious effect of T2D on LV remodeling and function in patients with severe AS, but so far, the underlying mechanisms remain unclear. Mitochondria play a central role in the regulation of cardiac energy metabolism.

**Objectives:**

We aimed to explore the mitochondrial alterations associated with the deleterious effect of T2D on LV remodeling and function in patients with AS, preserved ejection fraction, and no additional heart disease.

**Methods:**

We combined an in-depth clinical, biological and echocardiography phenotype of patients with severe AS, with (n = 34) or without (n = 50) T2D, referred for a valve replacement, with transcriptomic and histological analyses of an intra-operative myocardial LV biopsy.

**Results:**

T2D patients had similar AS severity but displayed worse cardiac remodeling, systolic and diastolic function than non-diabetics. RNAseq analysis identified 1029 significantly differentially expressed genes. Functional enrichment analysis revealed several T2D-specific upregulated pathways despite comorbidity adjustment, gathering regulation of inflammation, extracellular matrix organization, endothelial function/angiogenesis, and adaptation to cardiac hypertrophy. Downregulated gene sets independently associated with T2D were related to mitochondrial respiratory chain organization/function and mitochondrial organization. Generation of causal networks suggested a reduced Ca^2+^ signaling up to the mitochondria, with the measured gene remodeling of the mitochondrial Ca^2+^ uniporter in favor of enhanced uptake. Histological analyses supported a greater cardiomyocyte hypertrophy and a decreased proximity between the mitochondrial VDAC porin and the reticular IP3-receptor in T2D.

**Conclusions:**

Our data support a crucial role for mitochondrial Ca^2+^ signaling in T2D-induced cardiac dysfunction in severe AS patients, from a structural reticulum-mitochondria Ca^2+^ uncoupling to a mitochondrial gene remodeling. Thus, our findings open a new therapeutic avenue to be tested in animal models and further human cardiac biopsies in order to propose new treatments for T2D patients suffering from AS.

**Trial registration:**

URL: https://www.clinicaltrials.gov; Unique Identifier: NCT01862237.

**Graphical abstract:**

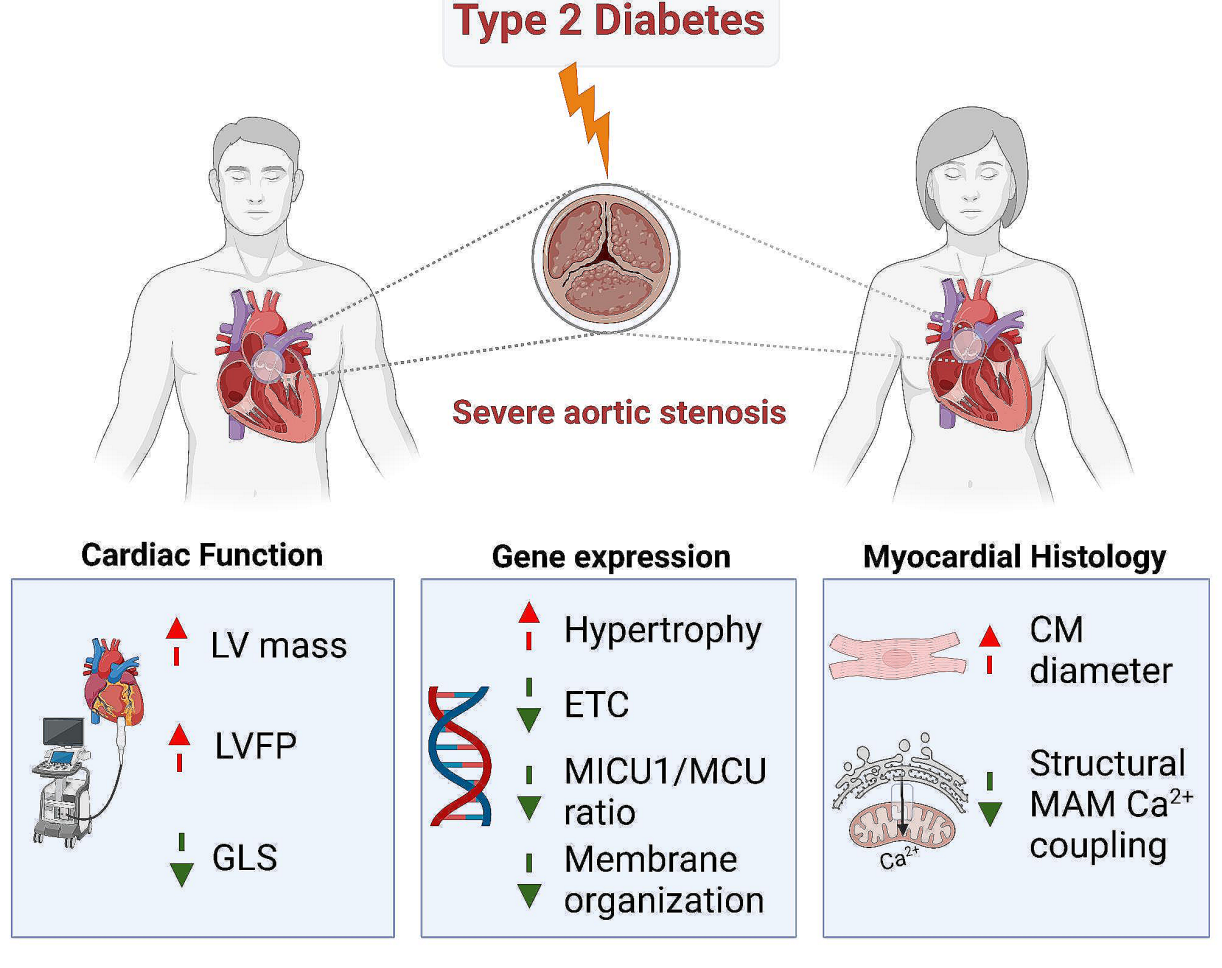

**Supplementary Information:**

The online version contains supplementary material available at 10.1186/s12933-024-02329-5.

## Background

Type 2 diabetes (T2D) is an increasingly prevalent health problem in Western societies, affecting at least 110 million people in Europe and North America [1], and expected to reach more than 10% of the worldwide population in 2045. Beyond its huge socio-economic burden, T2D increases the mortality mainly due to cardiovascular diseases [2]. T2D patients have twice as much risk of developing heart failure (HF) independently of the presence of coronary heart disease [3, 4]. Growing evidence has led to describing a specific cardiac phenotype called diabetic cardiomyopathy, which associates left ventricular (LV) concentric hypertrophy [5, 6] with systolic and diastolic dysfunction [7].

Aortic stenosis (AS) is the most common valvular heart disease in Europe and grows as the population gets older [8]. T2D is frequently associated with chronic LV pressure overload such as AS. The prognosis of severe AS becomes significantly worse after the onset of symptoms [9] but also when the LV displays an increased LV mass [10], a significant diastolic dysfunction [11] and/or a systolic LV dysfunction [12, 13]; alterations that are also associated with diabetic cardiomyopathy. Clinical studies have indeed suggested an aggravating effect of T2D on cardiac function in patients with severe AS [14], together with an increased mortality [15]. However, only few studies have assessed the underlying mechanisms of T2D-induced alterations in the context of AS. At pre-clinical level, we reported that insulin resistance induced by a high-fat diet in mice worsened survival, cardiac hypertrophy and systolic/diastolic dysfunction under aortic constriction [16]. In patients with AS, T2D has been associated to a higher cardiomyocyte resting tension, an increased vascular advanced glycation end product deposition [14], an impaired myocardial energetics as reflected by the reduced phosphocreatine to ATP ratio [17], oxidative stress and metabolic dysfunction [18]. Altogether, these data suggest a strong role for metabolic alterations in the deleterious effect of T2D on LV remodeling and function in patients with severe AS.

Mitochondria play a central role in the regulation of cardiac energy metabolism: mitochondrial Ca^2+^ concentration regulates the mitochondrial respiratory chain activity, ATP production and the activity of key mitochondrial dehydrogenases [19, 20], leading to the concept of the energetic regulation of the excitation–contraction coupling. Reticulum-mitochondria Ca^2+^ coupling (also called MAM for mitochondria-associated reticulum membranes) is also involved in the control of the excitation-energetics coupling by regulating mitochondrial Ca^2+^ handling [21, 22]. The mitochondrial Ca^2+^ uniporter (mtCU) represents the key structure which controls Ca^2+^ entry inside the mitochondrial matrix. A strategic positioning of the mtCU at the MAM interface has been demonstrated to enhance the excitation-energetics coupling [23]. Whether the reticulum-mitochondria Ca^2+^ coupling and the mtCU are altered by T2D in AS patients remains to be investigated, as more is needed to know about the underlying biology of diabetic cardiomyopathy in patients, given the paucity of human tissue harvesting.

In this context, we aimed to explore the mechanisms associated with the deleterious effect of T2D on LV remodeling and function in patients with severe AS, preserved EF, and no additional heart disease. To this end, we combined a deep echocardiography phenotype of patients with severe AS ± T2D referred for a valve replacement with transcriptomic and histological analyses of an intra-operative myocardial LV biopsy to test the hypothesis that the deleterious effect of T2D on cardiac remodeling during AS is associated with mitochondrial Ca^2+^ signaling alterations.

## Methods

### Trial design and patients

Consecutive patients with severe AS referred for surgical aortic valve replacement (AVR) were enrolled in a prospective bicentric (Lyon and Saint Etienne University Hospitals) cohort study called DIAPASON (Does type II dIAbetes influence Prognosis and/or left ventricular remodeling in patients with Aortic valve Stenosis referred for aOrtic valve replacemeNt, NCT01862237). The present study focused on preoperative analyses.

Patients addressed for aortic valve replacement were included, after informed consent, only when meeting the following criteria: age > 18 years, severe AS (Aortic orifice surface < 1 cm^2^ or < 0.6 cm^2^/m^2^), LVEF > 50%, absence of overt heart disease or regional LV wall motion abnormalities, good acoustic window, affiliation to the social security system and no legal protection. Patients were excluded if they have type I diabetes or hyperglycemia without diabetes (fasting glucose between 1.1 and 1.25 g/L), chronic arrhythmia, significant coronary stenosis, history of cardiomyopathy including myocardial infarction, a more than mild other valvular diseases (mitral valve disease or aortic regurgitation), kidney failure (CI < 30 mL/mn) or uncontrolled hypertension (> 180/100 mmHg).

Patients were considered T2D if under anti-diabetic medication and/or with fasting glucose ≥ 126 mg/dL (6.99 mmol/L). Control patients (non-diabetic patients) were defined by fasting blood sugar < 110 mg/dL (6.11 mmol/L) and without any anti-diabetic medication.

Clinical data were collected at the inclusion. A complete physical examination was performed, including height, weight, blood pressure, and heart rate measurements. Blood samples were taken for routine biochemical analysis of HbA1c, brain natriuretic peptide, creatinine levels and lipid profile, and for subsequent collection.

Resting transthoracic echocardiography was performed using commercially available ultrasound systems (Vivid E9 or E95, General Electric). Images were digitally stored.

A LV myocardial biopsy was collected from the septum base during the aortic valve surgery. The biopsy was cut in two halves: one was formalin-fixed for histological analyses, and the other was snap-frozen in liquid nitrogen for mRNA analyses. The sample size for each endpoint is listed in Supplemental Table 1.

### Measurements of biomarkers concentrations

Serum samples were stored at − 80 °C. The concentration level of different biomarkers was analyzed with enzyme-linked immunosorbent assay (ELISA) in serum with commercially available kits: insulin, leptin, FABP3, TIMP1, galectin 3, MMP9, S100B, Serpin, IL-6, ST2, CXCL9, CXCL10 (DuoSet ELISA by R&D Systems) and Endothelin 1 (QuantiGloTM ELISA).

### Echocardiography analysis

Echocardiographic studies were blindly analyzed offline (EchoPac, GE Vingmed) at the end of the inclusions.

Aortic valve stenosis severity was evaluated using mean aortic gradient, aortic valve (AV) area (AVA) based on the continuity-equation calculation [24] and the VTI ratio (VTI_LVOT_/VTI_AV_)_._

Valvuloarterial impedance (Zva), which represents the global systolic load imposed on the LV, was also estimated using the following formula: Zva^2.04^ = (mean aortic gradient + SBP)/(SV/height^2.04^).

Left ventricular remodeling was assessed following current guidelines [25].

Left ventricular end-diastolic and end-systolic volume (LVED and LVES) were calculated with the biplane Simpson method. Posterior wall thickness (PWT), interventricular septum thickness (IVST), and left ventricular end-diastolic diameter (LVEDD) were measured in parasternal long-axis views in 2D.

The left ventricular mass was assessed in 2D and 3D echocardiography. 2D left ventricular mass in grams (2D LVM) was calculated using the cube formula: 2D LVM = 0.8 × 1.04 ((IVST + LVEDD + PWT)3 − LVEDD3) + 0.6.

Three-dimensional LVM was measured on adequate apical acquisitions performed on 3 to 4 consecutive cardiac cycles (frame rate > 25) during a brief apnea. 3D LV mass = LV epicardial volume − LV endocardial volume × 1.05 = LV myocardial volume × 1.05. LV mass was further indexed to height to allometric power of 2.7 [26–28]*.*

The left ventricular systolic function was assessed using left ventricle ejection fraction (LVEF %) using the biplane Simpson method, and global Longitudinal Strain (GLS) was measured from the three standard apical views after optimizing image quality, maximizing frame rate, and minimizing foreshortening. If the regional tracking was suboptimal in more than two myocardial segments in a single view, the GLS value was not considered.

Diastolic function and LV filling pressure assessment followed current algorithm [29, 30]. Transmitral inflow was recorded using pulsed wave Doppler in the apical 4-chamber view to measure early (E) and late (A) mitral inflow velocities. Early diastolic velocity was assessed at the septal (septal e′) and lateral (lateral e′) sites of the mitral annulus using pulsed wave tissue Doppler imaging. The average value of e′ was used to calculate E/e′ ratio [31]. Left atrial volume LAVi was estimated by the biplane Simpson method and indexed to BSA and to height^2^ [32]. The atrial strain was assessed in 4 and 2 chamber view [33]. Left ventricular filling pressure was classified as normal, elevated, or indeterminate [30].

### Histological analysis of LV biopsies

Endomyocardial biopsies were fixed in 10% buffered formalin, paraffin-embedded, and cut at 4 µm for subsequent staining. Both qualitative and quantitative histological analyses were performed blindly.

#### Qualitative analysis

Myocyte hypertrophy, immune infiltration (HES) and fibrosis (Sirius Red) were graded blindly using a qualitative scale (mild, moderate, severe) by a cardiovascular pathologist.

#### Myocyte diameter

Wheat germ agglutinin immunostaining (ThermoFisher W11261, 0.02 mg/mL) was performed to manually measure the myocyte diameter at the nucleus level on Fiji (an image processing package, used with image J) using slides digitized at 40×.

#### Fibrosis quantification

A custom macro on Fiji was used to semi-automatically quantify the percentage of collagen stained in red on Sirius Red-stained sections.

#### TUNEL assay

Apoptosis was quantified with the TUNEL assay (Invitrogen C10618, AlexaFluor 594) following the manufacturer’s instructions. The total cell number and number of apoptotic cells were semi-automatically quantified using Fiji on 10× images.

#### Proximity ligation assay (PLA)

PLA was conducted to quantify the proximity between the reticular IP3R-receptor (ab5804, 1/20000) and the mitochondrial VDAC porin (ab14734, 1/40000), according to the manufacturer’s instruction (Sigma). Images were taken on a BX63 microscope at 60×. Quantification of the number of dots per cell was performed semi-automatically on Fiji.

### Method and statistical analyses of gene expression

#### RNA isolation and RNAseq

Samples were crushed into powder in liquid nitrogen. Total RNA extraction was performed using miRNeasy mini kit (Qiagen) following the manufacturer’s instructions. Purity and concentrations were measured using Nanodrop 2000 (Ozyme), while integrity was checked using Bioanalyser (Agilent). Fifty-five samples with ≥ 100 ng total RNA and DV200 > 30% were further processed for sequencing using Lexogen QuantSeq 3 mRNA seq Library Prep kit (Illumina). Two samples were excluded due to low library quality. Fifty-three libraries were sequenced on an Illumina NextSeq 500, generating around 8 million 75 bp single-end reads per library. Bcl2-fastq (v.2.17.1.14) was used for demultiplexing. After suppression of 3 samples due to high duplication level and of 3 outliers identified by principal component analysis, 47 samples were included in the RNAseq data analysis (Supplemental Fig. 1; Supplemental Table 2).

#### Analysis of differential gene expression

FastQC (v0.11.9) was used to check raw read quality. Adaptor removal was performed using Cutadapt (v4.0) with default parameters. Only 75 bp-long reads were kept. Good Phred scores (> 30) were obtained in all the libraries. However, three libraries showed high duplication levels leading to their exclusion. RNAseq reads’ alignment was performed using STAR (v2.7.5a) against the GRCHh38 reference genome. DEseq2 (v1.36.0) was used for differential gene expression identification starting from counts generated by STAR (v2.7.5a). PCA plot was performed in R using vast transformed counts for outlier identification. Three samples were excluded from the following analysis. Finally, data from forty-seven samples were used for Gene expression analysis after normalization using the DESeq2. Genes were considered differentially expressed using the 5% P value threshold for significance. Adjustment for covariates was performed using generalized linear models within DESeq2. Functional Enrichment analysis was determined with gProfiler using only the annotated genes from the Human genome as background gene set, and represented as networks using EnrichmentMap in Cytoscape 3.8.2. The detailed RNA-seq information is available in GSE236191 deposited in the NIH GEO database.

### Statistical analyses

Quantitative data were described using median, first and third quartiles and qualitative data were described using effectives, percentages. Non-parametric tests (Wilcoxon or Fisher exact test) were done to compare distribution between groups.

We used univariate and multivariate linear regressions to examine predictive factors of 3D LV mass index to height^2^ and Global Longitudinal Strain. We used univariate and multivariate logistic regressions to examine predictive factors of left ventricular filling pressure. Finals models were estimated using a backward stepwise procedure based on the minimization of the AIC criterion.

Statistical significance was defined as p-value < 0.05. All statistical analyses were performed using the R statistical Software version 3.3.3.

## Results

### Baseline characteristics of study patients

Aortic valve replacements were performed in 84 patients, including 34 with T2D. Baseline characteristics, including comorbidities and treatment of the population, are displayed in Table 1. The median age was 67 IQR [54; 75], and the proportion of enrolled women (38%) did not differ between the two groups. Hypertension (p < 0.01) and dyslipidemia (p < 0.01) coexisted more frequently in T2D patients. T2D patients had a greater BMI (p < 0.01) and BSA (p < 0.05). Renal function was normal and similar between groups (Supplemental Table 3).

**Table 1:** Pre-operative baseline characteristics

Variable	Non-diabetic n = 50	T2D n = 34	p Value
Age (years)	68 [61; 72]	67 [63; 74]	ns
Women (N, %)	19 (38%)	13 (38%)	ns
Medical history			
BMI	25 [24; 27]	29 [26; 32]	< 0.001
BSA (m^2^)	1.8 [1.7; 1.9]	1.9 [1.7; 2.1]	< 0.05
Hypertension (N, %)	20 (41%)	30 (88%)	< 0.001
Retinopathy	0 (NaN%)	3 (10%)	ns
Clinical evaluation			
NYHA grade (N, %)			< 0.01
Class I	16 (32%)	2 (5.9%)	
Class II	19 (38%)	14 (41.2%)	
Class III	14 (28%)	14 (41.2%)	
Class IV	1 (2%)	1 (2.9%)	
Unknow	0 (0%)	3 (8.8%)	
Blood pressure (mmHg)			
SBP	129 [118; 142]	137 [130; 148]	ns
DBP	72 [66; 78]	73 [66; 79]	ns
Risk factors			
Dyslipidaemia (N, %)	14 (28%)	23 (68%)	< 0.001
Smoker (N, %)	19 (38%)	15 (44%)	ns
Medications			
Insulin (N, %)	0 (0%)	10 (29%)	< 0.001
ACEI/ARB (N, %)	13 (26%)	22 (65%)	< 0.001
Betablockers (N, %)	8 (16%)	12 (35%)	ns
Statins (N, %)	13 (26%)	27 (79%)	< 0.001

Data are presented as n (%) or median [25th; 75th percentile]. p value displayed for Fisher exact test was used for categorical variables and the Wilcoxon test was used for continuous variables. *ns* not significant

*ACEI/ARB* ratio of angiotensin-converting enzyme inhibitor and angiotensin receptor blockers, *BMI* body mass index, *BSA* body surface area, *DBP* diastolic blood pressure, *SBP* systolic blood pressure

Most T2D patients were treated with oral antidiabetic drugs (24 with metformin or sulfonylureas). Ten patients (29%) with T2D received insulin. T2D patients were more frequently under ACEI/ARB and statins than non-diabetics. Only three T2D patients had diabetic retinopathy.

NYHA states differed between groups with more T2D patients in class III NYHA than non-diabetic patients. However, BNP levels were not significantly different (Table 1, Supplemental Table 3).

### T2D patients had similar AS severity but displayed worse cardiac remodeling and function than non-diabetics

Aortic mean gradient, VTI ratio, AVA, and cardiac index were similar between both groups. Valvuloarterial impedance and stroke volume index did not differ between groups (Table 2).

**Table 2:** Echocardiographic parameters

Variable	Non-diabetic	T2D	p Value
Aortic hemodynamic parameters			
Aortic mean gradient (mmHg)	51 [48; 64]	57 [47; 68]	ns
Aortic permeability index	0.20 [0.18; 0.23]	0.20 [0.19; 0.24]	ns
AVA (cm^2^)	0.72 [0.66; 0.82]	0.79 [0.64; 0.89]	ns
AVA indexed to BSA (cm^2^/cm^2^)	0.41 [0.36; 0.47]	0.39 [0.33; 0.49]	ns
AVA indexed to height^2.04^ (cm^2^/m^2.04^)	0.26 [0.23; 0.29]	0.26 [0.23; 0.32]	ns
SV (mL)	80 [73; 87]	75 [69; 91]	ns
Svi to BSA (mL/m^2^)	45.0 [40.1; 49.6]	41.1 [35.8; 48.2]	ns
Zva (mmHg/mL/m^2^)	4.2 [3.7; 4.9]	5.0 [3.8; 6.2]	ns
Zva^2.04^ (mmHg/mL/m^2.04^)	6.6 [6.0; 7.8]	7.7 [5.9; 9.2]	ns
Left ventricle remodeling			
IVS (mm)	15 [13; 16]	15 [14; 16]	ns
PWT (mm)	13 [11; 15]	13 [13; 16]	< 0.05
LVEDD (mm)	41 [36; 47]	41 [36; 45]	ns
EDV (BS) (mL)	94.5 [67.5; 113.5]	93.0 [74.8; 114.8]	ns
EDV (BS) (mL/m^2^)	49.9 [38.5; 62.5]	49.0 [40.4; 58.4]	ns
ESV (BS) (mL)	33.0 [23.3; 45.3]	35.5 [29.3; 46.0]	ns
ESV (BS) (mL/m^2^)	18.5 [13.2; 24.5]	19.3 [15.3; 24.1]	ns
3D LVM (g)	158 [133; 177]	169 [154; 221]	< 0.05
3D LVM/height^2.7^ (g/m^2.7^)	39 [33; 45]	46 [38; 52]	< 0.05
Systolic function			
LVEF %	64 [58; 69]	61 [57; 64]	< 0.05
GLS %	18 [17; 19]	15 [13; 17]	< 0.001
Mechanical dispersion	55 [46; 74]	61 [50; 74]	ns
LV radial strain (%)	54 [49; 61]	52 [39; 63]	ns
Diastolic function			
E/e′	12.0 [10.0; 15.0]	13.0 [11.9; 17.1]	ns
LA strain max (%)	21 [18; 31]	24 [17; 25]	ns
LA strain < 18%	6 (16%)	9 (38%)	ns
PAPS (mmHg)	31 [28; 35]	32 [29; 43]	ns
LAESV (mL)	60.5 [44.5; 83.3]	70.0 [62.0; 102.0]	< 0.01
LAESVI (mL/m^2^)	32.7 [25.6; 46.2]	38.7 [30.2; 51.8]	ns
LAEDVI (mL/m^2^)	18.4 [13.4; 29.3]	22.4 [17.8; 27.3]	ns
LAESVI/height^2^	22.1 [16.01; 29.7]	26.0 [20.8; 36.7]	< 0.05
LVFP			
Normal	33 (66%)	15 (47%)	ns
Indeterminate	4 (8%)	1 (3%)	ns
Elevated	13 (26%)	16 (50%)	< 0.05

Data are n (%) or median [25th; 75th percentile]. p value displayed for Fisher exact test was used for categorical variables and Wilcoxon test was used for continuous variables. *ns* not significant

*AVA* aortic valve area, *Sv* stroke volume, *Svi* stroke volume index, *Zva* valvuloarterial impedance, *Zva*^2.04^ valvuloarterial impedance with stroke volume index to height^2.04^, *EDV (BS)* end-diastolic volume (biplane Simpson), *ESV (BS)* end-systolic volume (biplane Simpson), *GLS* global longitudinal strain, *IVS* interventricular septal end diastole, *LAESV* left atrial end systolic volume, *LAEDVI* left atrial end diastolic volume index, *LAESVI* left atrial end systolic volume index, *LAESVI/height*^2.7^ left atrial end systolic volume index to height^2.7^, *LVEDD* left ventricular end-diastolic volume, *LVEF* left ventricular ejection fraction, *LVFP* left ventricular filling pressure (ref. 2022), *LVM* left ventricular mass, *PAPS* pulmonary artery systolic pressure, *PWT* posterior wall thickness

Considering LV remodeling, T2D patients displayed similar end-diastolic diameter and volume. T2D presented an increased LV hypertrophy with a thicker PWT (p < 0.05) and LV 3D mass (p < 0.05), as compared to non-diabetic patients.

T2D patients also presented a worse LV systolic function with a lower LVEF (61 [57; 64] vs 64 [58; 69] %, p < 0.05), nevertheless remaining preserved, and a lower global longitudinal strain (15 [13; 17] vs 18 [17; 19] %, p < 0.001) (Table 2).

Regarding diastolic function parameters, left atrial volume was larger in T2D patients, considering LAESVI and LAEDVI indexed to height (as recommended in overweight patients). PAPs and E/e′ ratio did not differ between both groups. LV atrial reservoir strain did not differ between groups but LV atrial reservoir strain tended to be more often < 18% in T2D patients. Half of the T2D patients had elevated LVFP compared to only 26% of the non-diabetic patients (p < 0.05, Table 2).

### T2D was independently associated with impaired systolic function

In the univariate analysis, including age, sex, hypertension, obesity, and AS severity, only BMI and T2D were associated with LV mass. In multivariate analyses, neither BMI nor T2D remained significantly associated with LV mass.

In the univariate analysis, only T2D was significantly associated with LV filling pressure, but not in the multivariate analysis.

In the univariate analysis, T2D and systolic arterial pressure were associated with GLS and only T2D remained independently associated with GLS in the multivariate analysis (Table 3).

**Table 3:** Multivariate analyses on LV mass, GLS and LV filling pressure

Parameter	Univariate B coef (95% CI)	p value	Multivariate B coef (95% CI)	p Value
3D LV mass index to height^2.7^				
T2D	5.57 (0.52–10.62)	< 0.05	0.9 (− 6.45–8.24)	ns
Age	0.12 (− 0.16–0.39)	ns		
Sex (women)	1.26 (− 3.71–6.23)	ns		
PAS	0.09 (− 0.05–0.23)	ns	0.07 (− 0.07–0.21)	ns
BMI	0.58 (0.02–1.14)	< 0.05	0.62 (− 0.3–1.54)	ns
Aortic mean gradient	0.1 (− 0.09–0.28)	ns		
Global longitudinal strain				
T2D	− 2.64 (− 3.73 to − 1.55)	< 0.001	− 2.04 (− 3.27 to − 0.8)	< 0.001
Age	− 0.01 (− 0.08–0.06)	ns		
Sex (women)	0.57 (− 0.65–1.8)	ns		
PAS	− 0.03 (− 0.06–0.01)	ns	− 0.02 (− 0.05–0.01)	ns
BMI	− 0.09 (− 0.23–0.05)	ns		
Aortic mean gradient	0 (− 0.04–0.05)	ns		
Left ventricular filling pressure				
T2D	2.71 (1.05–7.17)	< 0.05	1.96 (0.67–5.74)	ns
Age	1.04 (0.99–1.1)	ns	1.04 (0.99–1.1)	ns
Sex (women)	1.48 (0.58–3.82)	ns		
PAS	1.02 (0.99–1.04)	ns		
BMI	1.1 (1–1.23)	ns	1.07 (0.96–1.21)	ns
Aortic mean gradient	0.98 (0.94–1.02)	ns		

OR ratio used to binary variable and B coefficient for continued variable. Multivariate linear (B coef) and logistic (OR) regression with top-down stepwise procedure

*ns* not significant

### Increased levels of T2D circulating markers correlate with the T2D-induced cardiac remodeling and dysfunction in AS patients

**Fig. 1:**
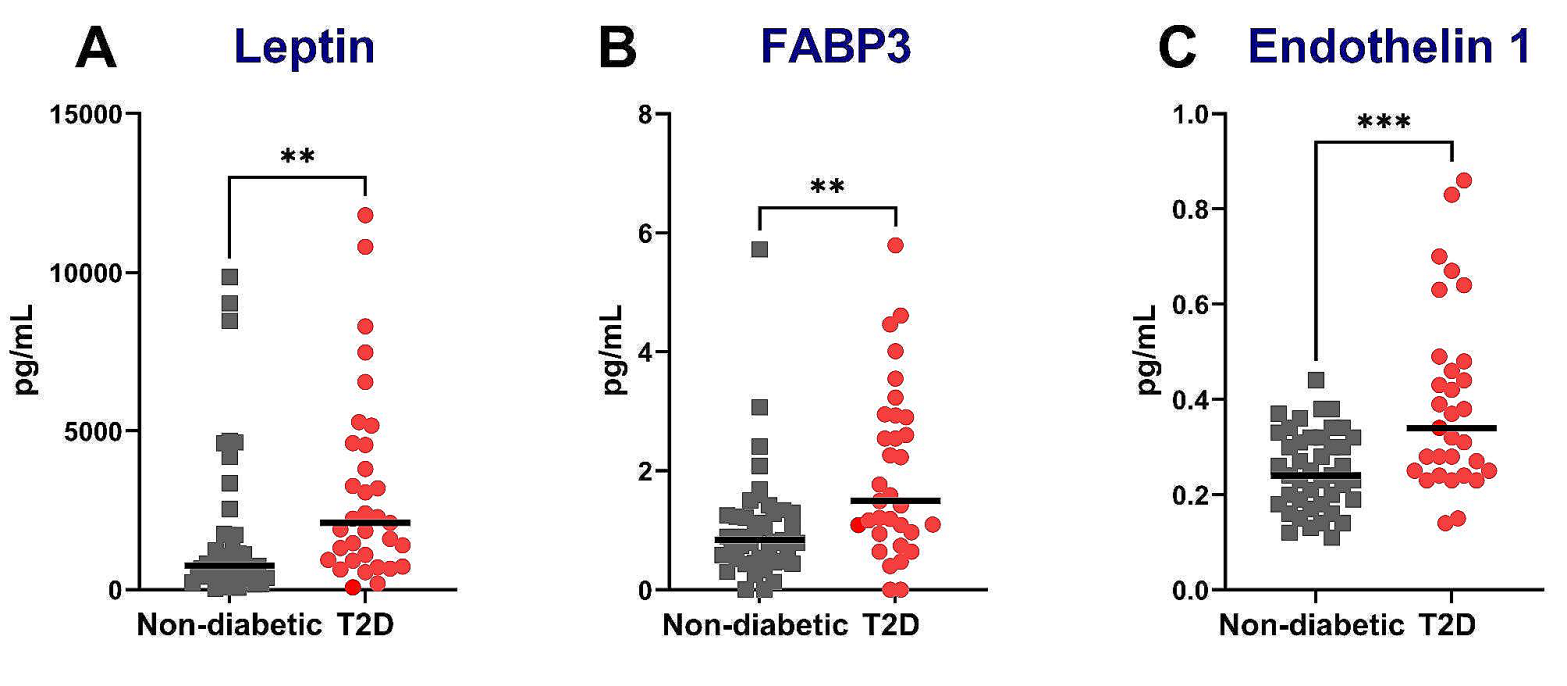
T2D increased circulating levels of metabolic markers in AS patients. Measurement of circulating serum biomarkers of diabetic cardiomyopathy focusing on leptin (**A**), FABP3 (**B**) and Endothelin 1 (**C**). Statistical analyses: Wilcoxon test. **p < 0.01; ***p < 0.001

We evaluated the serum circulating level of known markers of T2D. As expected, leptin as a mediator of long-term regulation of energy balance/food intake and FABP3, involved in lipid metabolism, were significantly increased in T2D compared to non-diabetic patients (Fig. 1A, B). Leptin level showed a modest but significant correlation with the diastolic dysfunction parameters (LA strain max r = − 0.23, p = 0.01; LAESVI r = 0.17, p < 0.05), and FABP3 with 3D LVM/height^2.7^ (r = 0.1057, p < 0.05).

Endothelin-1, a vasoconstrictor peptide, was significantly increased in the T2D group (Fig. 1C) and correlated with hypertrophy and systolic dysfunction (3D LVM/height^2.7^: r = 0.27, p < 0.001; GLS: r = − 0.25, p < 0.01).

Inflammatory and fibrotic circulating markers were not different between both groups (Supplemental Fig. 2).

### T2D triggers distinct transcriptomic myocardial signatures in AS patients

**Fig. 2:**
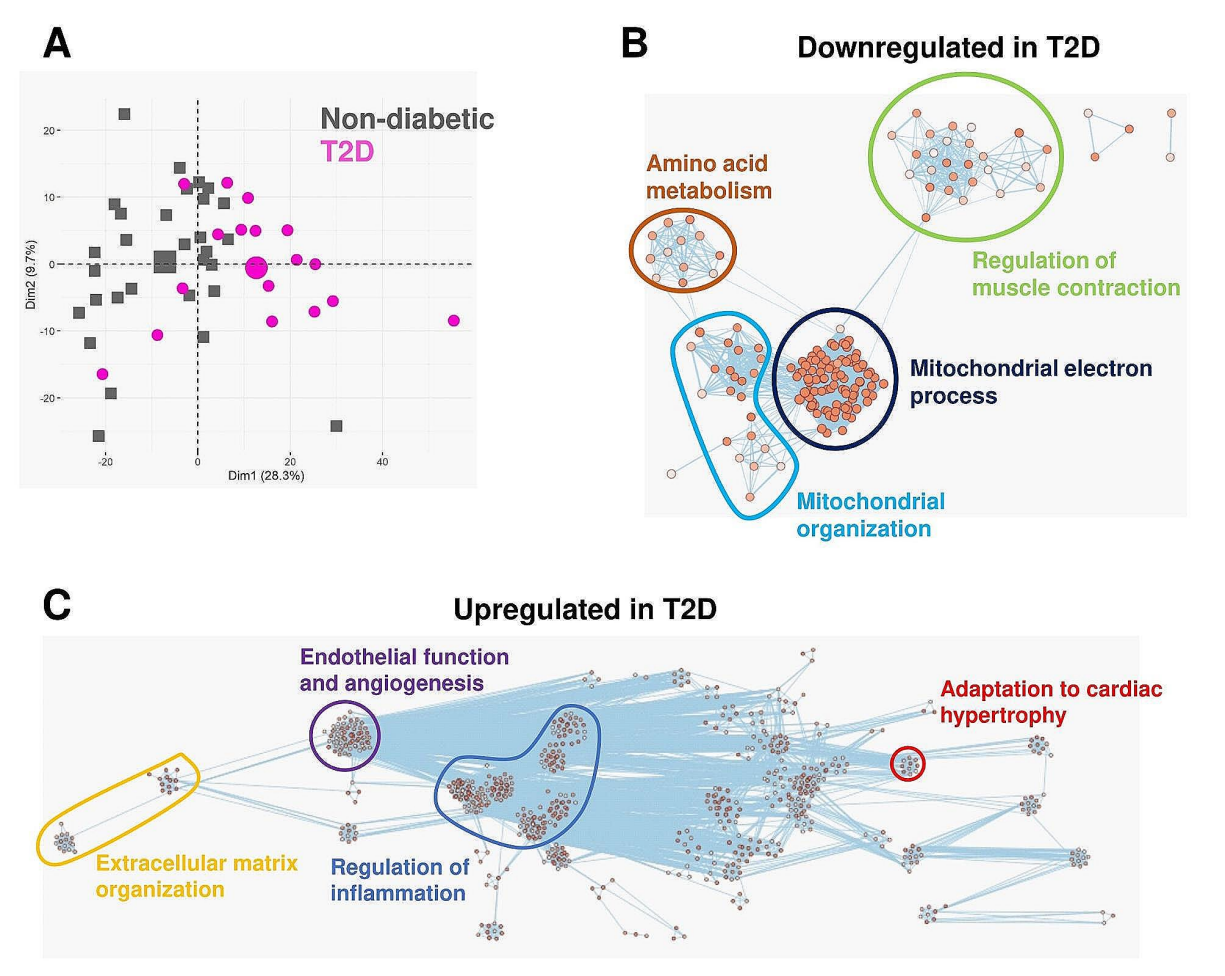
Myocardial transcriptomic changes induced by T2D in AS patients. RNAseq was performed on the myocardial biopsies. **A** Principal component analysis using the 1029 genes differentially expressed between non-diabetic (grey squares) and T2D (purple circles) patients. The bigger square and circle represent the gravity center for each cluster. **B**, **C** Enrichment maps displaying the significantly down- (**B**) or up-regulated (**C**) GO biological processes in T2D patients versus non-diabetics. Node color intensity is proportional to enrichment significance (Fisher exact P value after Benjamini-Hochberg (BH) adjustment for multiple comparisons). Relevant clusters of functionally related gene-sets were manually circled and assigned a label, based on suggested annotations by AutoAnnotate (Cytoscape)

The filtered RNAseq read-set identified 15,434 genes. Among them, 1029 genes were significantly differentially expressed between non-diabetic and T2D patients (Supplemental Table 4). Principal component analysis based on these 1029 genes revealed a segregation along the first dimension (PC1: 28.3%) between the non-diabetic and T2D patients (Fig. 2A). To identify which biological processes were affected, we performed an enrichment analysis focusing either on the up-regulated or the down-regulated genes. Only biological process GO annotations presenting an adjusted p value < 0.05 were considered as affected and were displayed on the enrichment maps (Fig. 2B, C).

Functional annotations significantly downregulated in T2D patients relate to mitochondrial respiratory chain organization and function, mitochondrial organization, amino acid metabolism, and regulation of muscle contraction (Fig. 2B).

The significantly upregulated pathways in T2D gather regulation of inflammation (leucocyte activation, neutrophil migration), extracellular matrix organization, endothelial function and angiogenesis, and adaptation to cardiac hypertrophy (Fig. 2C).

### Influence of T2D covariates on gene expression

**Fig. 3:**
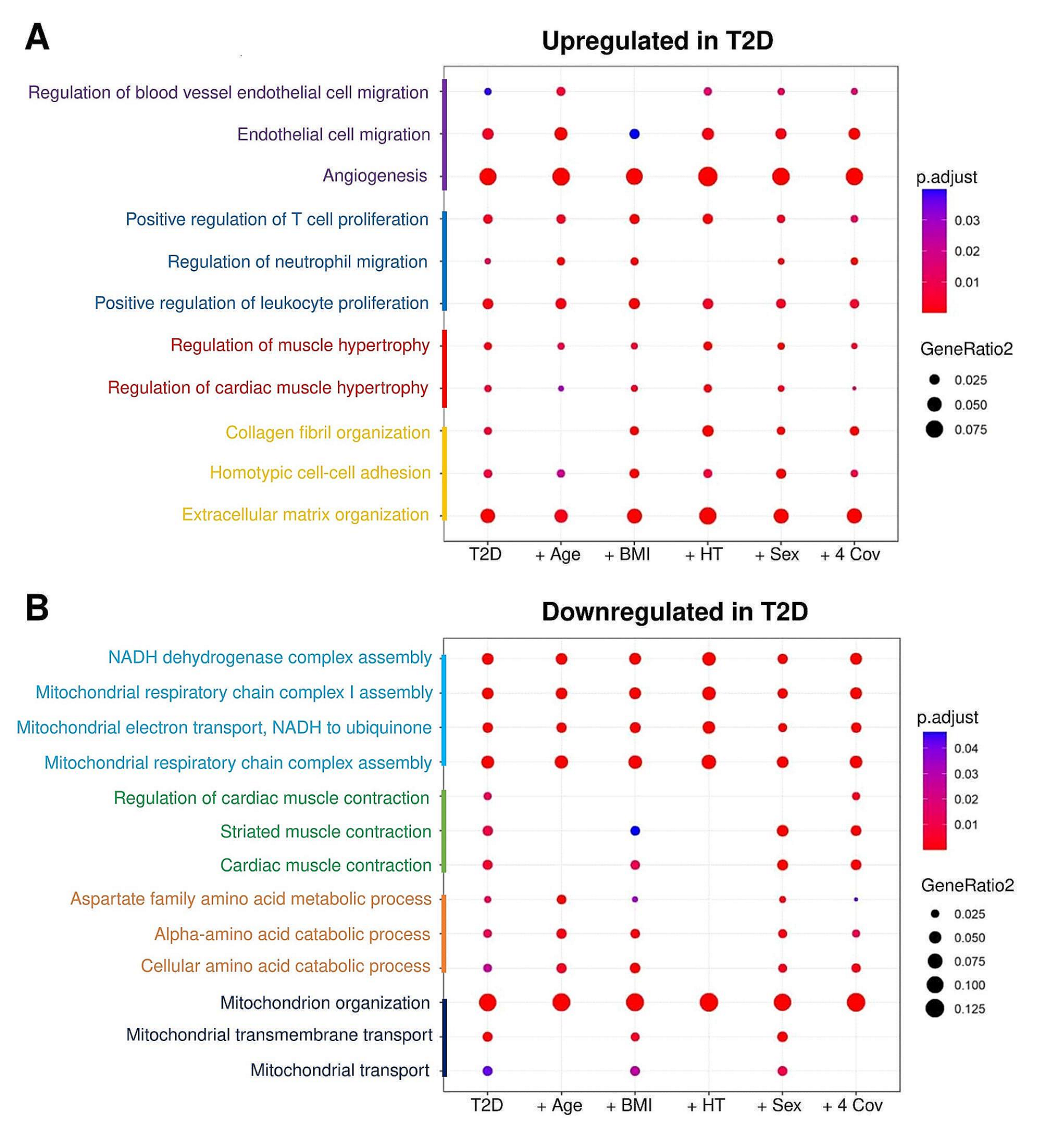
Effect of comorbidities on differentially expressed T2D genes. Bubble plots represent for each main cluster identified in Fig. 2B, C, the enrichment of the most differentially expressed GO biological processes either up- (**A**) or down-regulated (**B**) in T2D patients versus non-diabetics, after adjustment for age, BMI, hypertension (HT), sex or all the 4 covariates (4 Cov)

To test the influence of T2D covariates on gene expression changes, we used mixed linear models adjusting for age, BMI, hypertension, sex or all the 4. List of genes considered as differentially expressed were analyzed for biological pathways analysis. Regarding up-regulated genes, including covariates in the gene expression statistical model did not change the pathway enrichment (Fig. 3A). As for the downregulated gene pathways, the mitochondrial respiratory chain process and mitochondrial organization remained independently associated with T2D. However, the regulation of cardiac muscle contraction, the amino acid metabolism, and the mitochondrial transport, were no longer significantly associated with T2D after adjusting for covariates, mostly after adjustment with hypertension and age (Fig. 3B).

### Cardiac hypertrophy and mitochondrial dysfunction as main canonical signaling pathways induced by T2D

**Fig. 4:**
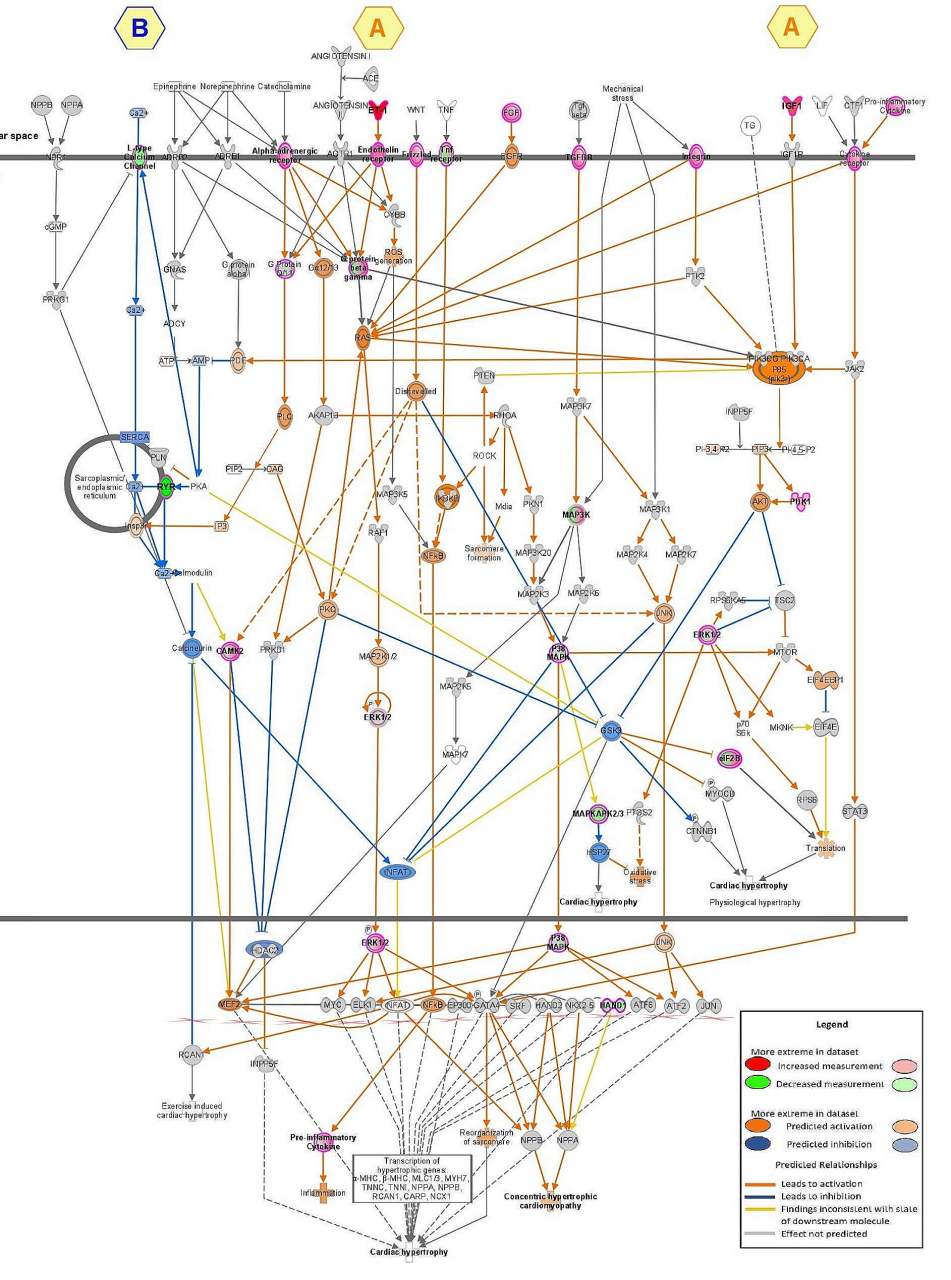
Cardiac hypertrophy signaling pathway in T2D myocardium identified by IPA. Relative changes in gene expression are depicted by gradated shades of color coding: upregulated in red; downregulated in green. Predicted relationships, i.e. activation in orange, inhibition in blue and inconsistency in yellow, are depicted by colored arrows

We next applied QIAGEN’s Ingenuity® Pathway Analysis (IPA) tool to our DEG dataset. Cardiac hypertrophy signaling appeared as an upregulated pathway (Fig. 4). Endothelin-1, its membrane receptor and IGF1 gene expressions were significantly upregulated by T2D, leading to a predicting activation of concentric hypertrophic cardiomyopathy pathways (Fig. 4, point A). In parallel, an inhibition of Ca^2+^ signaling was involved on this pathway (Fig. 4, point B), notably by a decreased gene expression of a L-type Ca^2+^ channel (CACNA1A**, **Table 4), and at the level of the sarco-endoplasmic reticulum with a decreased gene expression of ryanodine receptor (RyR) (Table 4), and a predicted inhibition of both the sarco-endoplasmic reticulum Ca^2+^ ATPase (SERCA) and the calmodulin gene expression.

**Table 4:** List of differentially expressed genes involved in mitochondrial signaling in T2D patients

Protein name	Log2 Fold change	p Value
OXPHOS—Complex I		
NDUFB9	− 0.306	< 0.01
NDUFS2	− 0.257	< 0.05
NDUFS6	− 0.274	< 0.05
NDUFV3	− 0.324	< 0.05
NDUFA13	− 0.463	< 0.01
NDUFB2	− 0.345	< 0.01
MT-ND1	− 0.322	< 0.05
MT-ND2	− 0.216	< 0.05
MT-ND3	− 0.461	< 0.01
MT-ND4L	− 0.488	< 0.01
OXPHOS—Complex III		
UQCR10	− 0.276	< 0.05
UQCR11	− 0.319	< 0.05
OXPHOS—Complex IV		
COX7C	− 0.322	< 0.05
COX4I1	− 0.251	< 0.05
OXPHOS—Complex V		
ATP5PF	− 0.234	< 0.05
ATP5MJ	− 0.298	< 0.01
ATP5MG	− 0.241	< 0.05
ATP5ME	− 0.284	ns
ATP5PO	− 0.184	ns
Reticulum-mitochondria coupling		
ITPR3	0.102	ns
ITPR2	0.054	ns
ITPR1	0.123	ns
VDAC1	− 0.089	ns
VDAC2	− 0.097	ns
VDAC3	− 0.165	ns
MFN2	− 0.160	ns
HSPA9 (GRP75)	− 0.064	ns
RYR2	− 0.373	< 0.05
SERCA2	− 0.051	ns
CACNA1A (L type Ca^2+^ channel)	− 0.436	< 0.05
Mitochondrial calcium uniporter		
MCU	0.413	< 0.01
MCUB	0.465	< 0.05
MICU1	− 0.253	ns
MICU2	− 0.049	ns
MICU3	− 0.324	< 0.01
SMDT1 (EMRE)	− 0.195	ns
Permeability transition		
SLC25A4 (ANT1)	0.043	ns
SLC25A5 (ANT2)	− 0.040	ns
SLC25A6 (ANT3)	− 0.221	< 0.05
PPIF (Cyp D)	− 0.302	ns

*ns* not significant

**Fig. 5:**
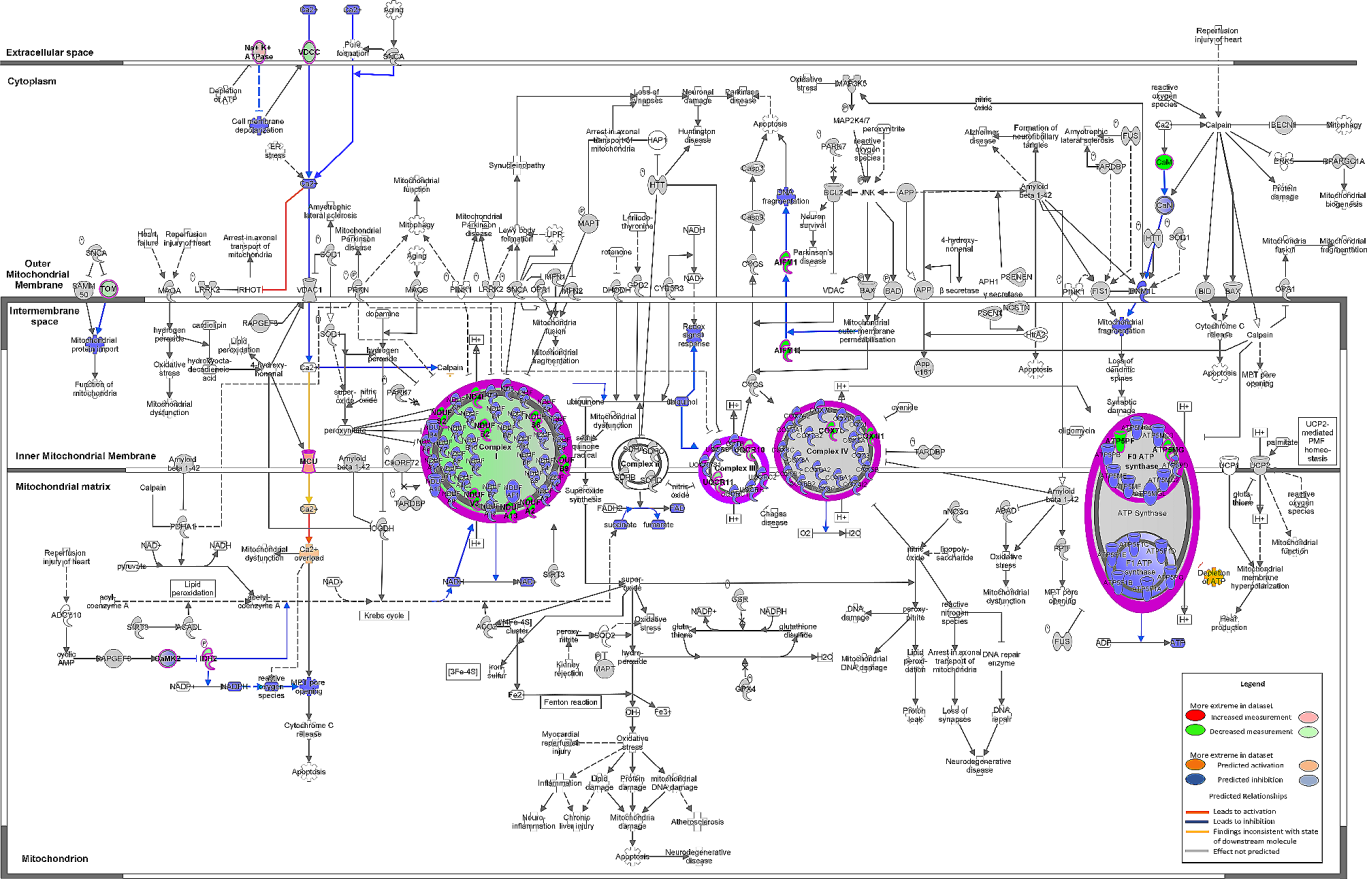
IPA analysis of mitochondrial signaling pathway alteration induced by T2D. Relative changes in gene expression are depicted by gradated shades of color coding: upregulated in red; downregulated in green. Predicted relationships, i.e. activation in orange, inhibition in blue and inconsistency in yellow, are depicted by colored arrows

Additionally, canonical signaling pathway analysis by IPA revealed a downregulation of mitochondrial signaling by T2D (Fig. 5). Several genes were significantly downregulated for complexes I, III and IV of the respiratory chain, as well as in the ATP synthase (Table 4). In parallel, a modification of mitochondrial Ca^2+^ signaling is displayed (Fig. 5). A decreased gene expression of a voltage-dependent calcium channel (CACNA1A, a L type Ca^2+^ channel, Table 4) is observed at the plasma membrane, predicted to decrease Ca^2+^ signaling up to mitochondria. Among the porins in the outer mitochondrial membrane, only VDAC3 tended to display a gene expression reduction by T2D. Concerning the mtCU in the inner mitochondrial membrane, both the pore-forming protein MCU and its negative dominant isoform MCUb were significantly increased at the gene level in T2D myocardium, while its regulatory proteins, MICU1 and MICU3, were downregulated (Table 4). Altogether, these changes led to a reduced MICU1/MCU ratio (1.77 [1.03; 2.26] vs 2.37 [1.68; 3.46], p < 0.05) and an increased MCU/EMRE ratio (0.30 [0.23; 0.41] vs 0.23 [0.16; 0.28], p < 0.01) without significant change in the MICU1/MICU2 and MCU/MCUb ratios. This is associated with an annotated “inconsistent finding”: the mtCU gene remodeling (reduced MICU1/MCU ratio) is predicted to increase the mitochondrial Ca^2+^ uptake [34]. However, since the cytosolic Ca^2+^ signaling is predicted to be decreased, the increased mitochondrial Ca^2+^ uptake may partially compensate for the reduced mitochondrial Ca^2+^ signaling, therefore leading to a predicted inhibition of permeability transition (Fig. 5) with a significantly reduced gene expression of ANT3 and components of ATP synthase, as proposed constituents of the permeability transition pore [35], while its regulator Cyclophilin D tended to be increased (Table 4).

### Tissue validation of T2D-induced cardiomyocyte hypertrophy, apoptosis and structural reticulum-mitochondria Ca^2+^ uncoupling

**Fig. 6:**
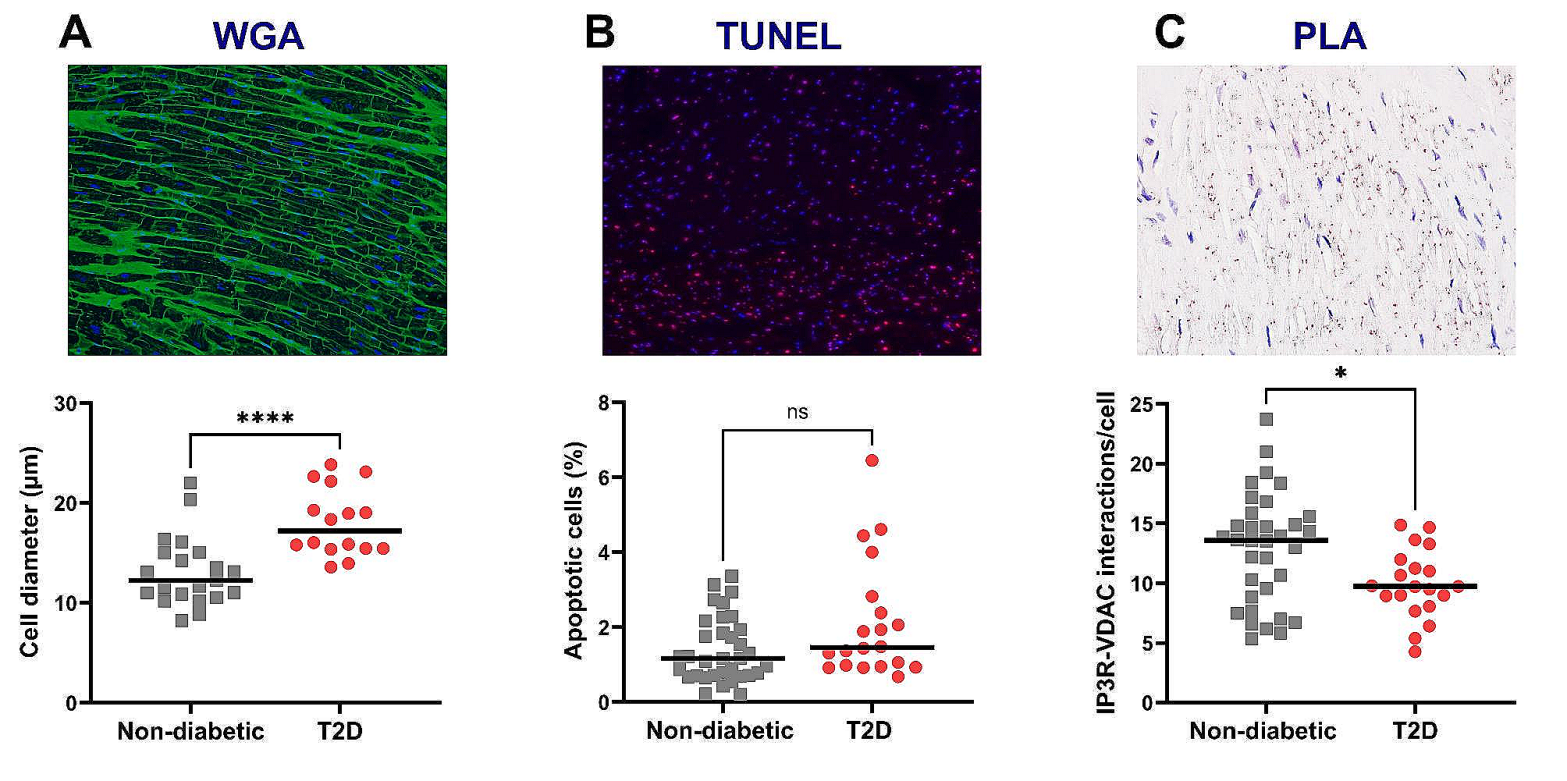
Histological validation of myocardial alterations induced by T2D in AS patients. **A** Measurement of cardiomyocyte diameter, an index of cell hypertrophy. Upper: representative image of WGA staining. Lower l: quantification of cell diameter. Magnification: 40×. **B** Analysis of apoptosis. Upper: typical image of TUNEL assay, showing nuclei in blue and apoptotic cells in red. Lower: quantification of the percentage of apoptotic cells. Magnification: 10×. **C** Proximity ligation assay to evaluate the proximity between the reticular IP3-receptor and the mitochondrial porin VDAC. Upper: representative picture of PLA between IP3R and VDAC. Nuclei in blue and IP3R-VDAC interactions in red. Lower: quantification of the number of interactions between IP3R and VDAC per cell. Magnification: 60×. Statistical analyses: Fisher and Wilcoxon tests. *ns* not significant; *p < 0.05; ****p < 0.0001

To assess the relevance of the altered signaling pathways identified at the gene level, we performed a histological analysis on LV myocardium from patients of both groups. Qualitative evaluation of cardiomyocyte hypertrophy on H&E-stained sections revealed hypertrophy in both groups with more grade 2–3 (severe hypertrophy) in T2D patients (Supplemental Fig. 3A, B). Quantitative measurements by WGA staining showed an increased cardiomyocyte diameter in the T2D LV myocardium compared to controls (17 [15; 2] vs. 12 [11; 15] µm, p < 0.001, Fig. 6A).

TUNEL assay showed a tendency of an enhanced proportion of apoptotic cells in the T2D myocardium compared to non-diabetic ones (1.46 [0.98; 2.49] vs. 1.17 [0.71; 1.9] %, p = 0.051, Fig. 6B).

The extent of fibrosis and inflammatory cell infiltration appeared similar between both groups at this stage, by either qualitative or quantitative analyses (Supplemental Fig. 3C–F).

Assessment of structural Ca^2+^ coupling between the reticulum and mitochondria, at the level of the VDAC/Grp75/IP3R Ca^2+^ channeling complexes involved in regulation of ATP production in cardiomyocytes [36], displayed a significant reduction in the number of IP3R-VDAC interactions in the diabetic myocardium (10 [9; 11] vs. 14 [9; 16] interactions per cell, p < 0.05, Fig. 6C). Interestingly, the gene levels of IP3Rs, Grp75 and the MAM tether MFN2 were not changed by T2D (Table 4).

## Discussion

**Fig. 7:**
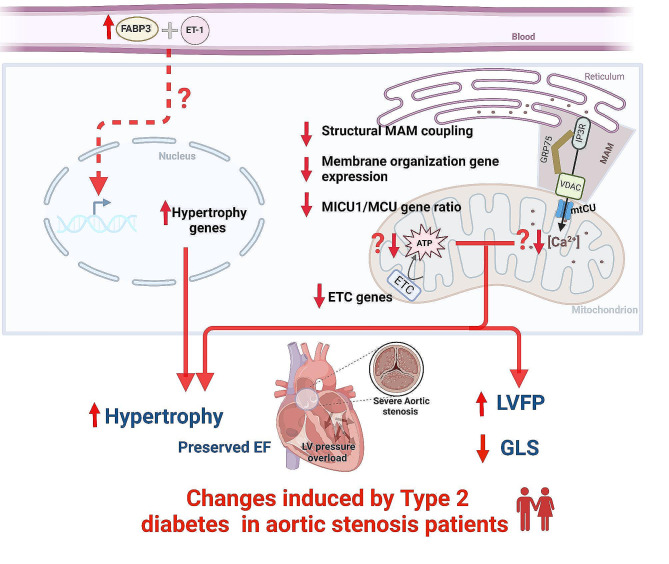
Central scheme depicting the T2D-specific biological processes associated with T2D-induced left ventricular dysfunction in patients with severe aortic stenosis. *ETC* electron transport chain, *ET-1* endothelin-1, *FABP3* fatty-acid binding protein 3, *GLS* global longitudinal strain, *LVFP* left ventricular filling pressure, *MAM* mitochondria-associated membranes

Combining RNA sequencing of LV biopsies from patients with severe AS, to a characterization of the non-invasive cardiac function allowed us to identify adverse LV remodeling and unique biological pathways associated with T2D during AS (Fig. 7). Our results strengthened the key role of mitochondrial calcium signaling in the diabetic human myocardium under chronic pressure overload, notably a reticulum-mitochondria structural Ca^2+^ uncoupling and a remodeling of the mtCU composition at gene level, paving the way for more focused functional studies to find potential therapeutic targets to limit the deleterious effect of T2D on cardiac function and remodeling in patients with severe AS.

### Mitochondrial dysfunction

The normal heart displays a metabolic flexibility regarding its substrate choice: mainly fatty acid oxidation, followed by glucose oxidation, requiring the TCA cycle and the oxidative phosphorylation as final steps. Several preclinical studies support a loss of metabolic flexibility in the diabetic heart, which remains mainly dependent on fatty acid oxidation [37]. In the context of AS, an association between altered myocardial energetics, i.e. reduced PCr/ATP ratios, and contractile dysfunction have been reported in T2D patients [17, 38, 39]*.* However, the underlying molecular mechanisms leading to this reduced ATP production in the human diabetic heart under chronic pressure overload remained unclear. Our transcriptomic analyses unraveled, in the context of AS, a significant T2D-specific downregulation of genes involved in mitochondrial respiratory chain assembly and mitochondrial membrane organization, that could account for the reduced mitochondrial ATP production via oxidative phosphorylation. Interestingly, T2D patients without AS have been shown to display impaired myocardial energetics [38, 39] and reduced mitochondrial respiration [40], thus suggesting the altered oxidative phosphorylation as a key dysfunction in the diabetic human heart, independently of additional disease.

Mitochondrial Ca^2+^ level is also crucial for the functioning of several matrix dehydrogenases. The structural reticulum-mitochondria Ca^2+^ uncoupling measured in the human T2D myocardium under severe AS may contribute to the reduced Ca^2+^ transfer to mitochondria and the subsequent alteration of mitochondrial Ca^2+^ level and energetics. In this regard, a murine model of diabetic cardiomyopathy with preserved EF displayed a decreased reticulum-mitochondria Ca^2+^ coupling as a critical trigger of cardiac dysfunction concomitantly to an increased cytosolic Ca^2+^ level [41]. Interestingly, at gene level, we measured a decreased expression of both L-type Ca^2+^ channel and RyR in the human diabetic myocardium of AS patients: this decrease could be considered as an attempt to counteract the increased cytosolic Ca^2+^ level in T2D hearts. Our RNAseq analysis also unraveled an increased MCU level and a trend towards a decreased MICU1 expression, leading to a reduced MICU1/MCU ratio, potentially leading to alteration of the mtCU protein composition. A lower MICU1/MCU ratio has been shown to reduce the mtCU threshold [34] and favor mitochondrial Ca^2+^ uptake and ultimately overload [42]. An increased MICU1/MCU protein ratio has been demonstrated in human end-stage failing hearts, as a possible maladaptation to the HFrEF-induced mitochondrial Ca^2+^ overload [43]. Therefore, in the myocardium of T2D patients with severe AS, the reduced mitochondrial Ca^2+^ level and ensuing energetics due to the reticulum-mitochondria Ca^2+^ uncoupling, may be in part compensated by the remodeling of the mtCU at the gene level, i.e. a reduced MICU1/MCU ratio favoring the increased mitochondrial Ca^2+^ uptake. However, to avoid that this compensation becomes maladaptive over time and triggers mitochondrial Ca^2+^ overload and cell death, the measured increase in MCUb gene expression and decrease in MCU/EMRE ratio may act as safekeepers of a maladaptive compensatory remodeling of the mtCU.

Collectively, our study reveals alterations both at the level of the respiratory chain and of the mitochondrial Ca^2+^ signaling in the diabetic myocardium of patients with AS, as potential main contributors to the cardiac metabolic remodeling observed during diabetic cardiomyopathy [44].

### Cardiac hypertrophy

In our study of patients with severe AS but no other heart disease, we found that participants with T2D had a more pronounced LV hypertrophy than non-diabetic patients on echocardiography (LV concentric hypertrophy), which was further confirmed at the cellular level by an enhanced cardiomyocyte diameter. In the particular setting of AS, Paget et al. specifically addressed the impact of metabolic syndrome on LV remodeling in patients with mild to moderate AS but did not perform any tissue analysis as patients were not subjected to surgery. They reported an independent association between metabolic syndrome and LV concentric hypertrophy and dysfunction. In our study, in univariate but not in multivariate analysis, both T2D and BMI were associated with 3D LV mass. Therefore, it is possible that visceral obesity also participates to this increased LV hypertrophy. In previous works, FABP3 has been related to the control of cardiac insulin resistance [45] and fatty acid uptake [46], but also as an independent predictor for the occurrence of all-cause and CV mortality in T2D patients with HF [47]. In our study, the fatty acid binding protein FABP3 displayed significantly increased serum concentration in the T2D group and, interestingly, correlated positively with the 3D LV mass.

Concomitantly to a significantly increased cardiomyocyte hypertrophy in the T2D myocardium, our transcriptomic analysis revealed an upregulation of genes involved in regulating cardiac muscle hypertrophy specifically driven by T2D. ET-1 was increased at both circulating and myocardial gene levels in T2D patients, and correlated with the increased heart weight. Canonical pathway analysis indeed supports an activation of the concentric cardiac hypertrophy signaling pathway by ET-1 in the diabetic myocardium from AS patients.

Mitochondrial dysfunction has also been linked to cardiac hypertrophy in several animal models [48]. A multi-omics study in patients with hypertrophic cardiomyopathy, including RNAseq, suggested defective ATP production as the primary contributor of hypertrophy [49]. Our data also support a critical role for the mitochondrial oxidative phosphorylation in cardiac hypertrophy since genes involved in respiratory chain formation were specifically downregulated in T2D patients. Additionally, single-cell transcriptional analysis revealed a decreased number of genes involved in the formation of the MAM in cardiomyocytes during the time course of hypertrophy induced by TAC in mice [50]. A reduced co-immunoprecipitation between IP3R and VDAC was reported in the hypertrophic mouse heart under TAC conditions [51]. Our histological analysis showed fewer interactions between IP3R and VDAC in the myocardium from T2D patients with severe AS, supporting a structural reticulum-mitochondria Ca^2+^ uncoupling that may also play a role in triggering cardiac hypertrophy.

Altogether, our findings advocate for a T2D effect on cardiac hypertrophy in patients with pressure overload, going from gene regulation to altered cellular architecture.

### Study limitations

In our study, we could not match patients based on comorbidities due to the goal of the study, i.e. evaluate the impact of T2D on severe AS; but patients were matched on demographics. A T2D effect still remained after comorbidity adjustment, for left ventricular systolic function (GLS) and the transcriptomic signature. Although both groups did not display the same number of patients, one strength of our study is to have a relatively large number of patients in each group, with all the measurements, from clinical to molecular, performed on almost all the patients, therefore allowing a comprehensive analysis of the LV function structure and molecular composition. As we did not perform single-cell RNAseq, changes in cellular composition may have caused a bias in our analysis. Moreover, the transcriptomic analysis does not necessarily reflect protein changes, but is of great relevance to digging out the underlying mechanisms.

Thus, further studies are needed to decipher the functional changes in a new cohort with fresh cardiac biopsies. Nevertheless, we also performed LV histological analyses, which complemented the gene expression changes with structural data, notably the reticulum-mitochondria Ca^2+^ uncoupling and the cardiomyocyte hypertrophy.

## Conclusions

Collectively, our data—from cardiac imaging phenotyping to tissue analysis—strongly support a crucial role for mitochondrial Ca^2+^ signaling in T2D-induced cardiac dysfunction during severe AS. We reported a structural reticulum-mitochondria Ca^2+^ uncoupling together with a remodeling of the mtCU composition at gene level in the LV of AS patients with T2D and preserved EF: importantly, these alterations may not be confined to pressure-overload patients and thus be encountered in patients with diabetic cardiomyopathy evolving towards metabolic HFpEF. With the recent development of screening strategies to identify new mtCU modulators [52] or to repurpose FDA-approved drugs [53], restoration of mitochondrial Ca^2+^ signaling opens a new therapeutic avenue to be tested in animal models and further human cardiac biopsies in order to propose new treatments for T2D patients suffering from AS.

### Supplementary Information

Supplementary material

## Data Availability

The datasets generated and analysed during the current study are available in the repository GSE236191 deposited in the NIH GEO database (https://www.ncbi.nlm.nih.gov/geo/query/acc.cgi?acc=GSE236191).

## Abbreviations

ANT3: Adenine nucleotide translocase 3
AS: Aortic stenosis
ATP: Adenosine triphosphate
AV: Aortic valve
AVA: Aortic valve area
AVR: Aortic valve replacement
BMI: Body mass index
BNP: Brain natriuretic peptide
BSA: Body surface area
Ca^2+^: Calcium
CACNA1A: Calcium voltage-gated channel subunit alpha 1A
CXCL9: Chemokine (C-X-C motif) ligand 9
EF: Ejection fraction
ELISA: Enzyme-linked immunosorbent assay
EMRE: Essential MCU regulator
ET-1: Endothelin-1
FABP3: Fatty acids binding protein 3
GLS: Global longitudinal strain
GO: Gene ontology
Grp75: 75-kDa glucose-regulated protein
HbA1c: Glycated hemoglobin
HES: Hematoxylin eosin safran
HF: Heart failure
HFpEF: Heart failure with preserved ejection fraction
HFrEF: Heart failure with reduced ejection fraction
IL-6: Interleukin 6
IP3R: Inositol 1,4,5-triphosphate receptor
IPA: Ingenuity pathway analysis
IQR: Interquartile range
IVST: Interventricular septal thickness
LAEDVI: Left atrial end diastolic volume index
LAESVI: Left atrial end systolic volume index
LAVi: Left atrial volume index
LV: Left ventricular
LVED: Left ventricular end diastolic
LVEDD: Left ventricular end diastolic diameter
LVEF: Left ventricular ejection fraction
LVES: Left ventricular end systolic
LVFP: Left ventricle filling pressure
LVM: Left ventricular mass
MAM: Mitochondria-associated reticulum membranes
MCU: Mitochondrial calcium uniporter
MCUb: Mitochondrial calcium uniporter b
MFN2: Mitofusin 2
MICU1: Mitochondrial calcium uptake family member 1
MMP9: Metalloproteinase 9
mRNA: Messenger ribonucleic acids
mtCU: Mitochondrial calcium uniporter
NYHA: New York heart association
PAPs: Pulmonary artery pressure
PC1: Principal component 1
PCA: Principal component analysis
PCr/ATP: Phosphocreatine/adenosine triphosphate ratio
PLA: Proximity ligation assay
PWT: Posterior wall thickness
RNAseq: Ribonucleic acid sequencing
RyR: Ryanodine receptor
S100B: Small calcium binding protein B
SBP: Systolic blood pressure
ST2: Suppression of tumorigenicity 2
SV: Systolic volume
T2D: Type 2 diabetes
TCA: Tricarboxylic acid cycle
TIMP1: TIMP metallopeptidase inhibitor 1
TUNEL: Terminal deoxynucleotidyl transferase dUTP nick end labeling
VDAC: Voltage-dependent anion channel
VTI: Velocity time integral
WGA: Wheat germ agglutinin
Zva: Valvuloarterial impedance
