# Supplementary material for: Myocardial transcriptomic analysis of diabetic patients with aortic stenosis: key role for mitochondrial calcium signaling
Source: Cardiovasc Diabetol. 2024 Jul 8;23:239. doi: 10.1186/s12933-024-02329-5 (PMC11232229; doi:10.1186/s12933-024-02329-5)
Supplement: Supplementary file 1 — Supplementary material [file 12933_2024_2329_MOESM1_ESM.docx]

**SUPPLEMENTAL MATERIAL**

**Myocardial transcriptomic analysis of diabetic patients with aortic stenosis: key role for mitochondrial calcium signaling**

**Authors:**

Maelle CHERPAZ, MSc,*^a,b^, Emmanuelle MEUGNIER, PhD,*^b^, Gaultier SEILLIER, MD,*^a^, Matteo POZZI, MD, ^a^ Romain PIERRARD, MD, ^c^, Simon LEBOUBE, MD, ^a,b^ , Fadi FARHAT, MD, ^e^ , Marco VOLA, MD,^e^ , Jean-François OBADIA, MD,^e^ , Camille AMAZ, PhD,^f^ , Lara CHALABREYSSE, MD, PhD,^d^, Chloe MAY, MSC,^f^, Stephanie CHANON, MSc,^b^, Camille BRUN, MSc,^b^ Lucas GIVRE^b^, Gabriel BIDAUX, PhD,^b^ , Nathan MEWTON, MD,^b,f^, Genevieve DERUMEAUX, MD, PhD,^a,g^ , Cyrille BERGEROT, MD, ^a,b^, Melanie PAILLARD, PhD,^#b^ & Helene THIBAULT, MD, PhD,^#a,b^

**Affiliations:**

^a^ Explorations Fonctionnelles Cardiovasculaires, Hospices Civils de Lyon, 69500 Bron, France

^b^ Laboratoire CarMeN - IRIS Team, INSERM, INRA, Université Claude Bernard Lyon-1, Univ-Lyon, 69500 Bron, France

^c^ Service de Cardiologie, CHU Nord, 42100 Saint-Étienne, France

^d^ Laboratoire d’anatomopathologie, Hospices Civils de Lyon, 69500 Bron, France

^e^ Chirurgie cardiaque, Hospices Civils de Lyon, 69500 Bron, France

^f^ Centre d’investigation clinique, Hospices Civils de Lyon, 69500 Bron, France

^g^ Current: INSERM U955, Université Paris-Est Créteil, Créteil, France ; AP-HP, Department of Physiology, Henri Mondor Hospital, FHU SENEC, Créteil, France

*These authors share the first authorship.

^#^ These authors share the last authorship and the correspondence.

**SOURCES OF FUNDINGS**

This project was funded by the French Ministry of Health and Research National Program (n° IR-27-24) and a grant from the Société Française de Cardiologie (Bourse Cœur, Vaisseaux, Diabète 2011) to HT, and by a grant from the Fondation de France (n°00107048) and a grant from the Agence Nationale de la Recherche (ANR- 20-CE14-0013-01) to MPa.

**DISCLOSURES**

None

**Correspondence:**

Pr Helene THIBAULT [helene.thibault@chu-lyon.fr](mailto:helene.thibault@chu-lyon.fr)

Dr Melanie PAILLARD [melanie.paillard@univ-lyon1.fr](mailto:melanie.paillard@univ-lyon1.fr)

Groupement Hospitalier Est

Service des Explorations Fonctionnelles & Laboratoire CarMeN

59 Boulevard Pinel, 69500 Bron – FRANCE

Tel: +33 (0)4.78.78.56.10

Fax number: +33 (0)4.72.35.69.10

Twitter: @PaillardMel

**#type2diabetes #aorticstenosis**

Page 3: Supplemental Table 1

Page 4: Supplemental Table 2

Page 5: Supplemental Table 3

Page 6: Supplemental Figure 1

Page 7: Supplemental Figure 2

Page 8: Supplemental Figure 3

**Supplemental Table 1**: **Sample size for each clinical and biological analysis**

|  | Non-diabetic | T2D | Total |
| --- | --- | --- | --- |
| Echocardiogram | **50** | **32** | **82** |
| Biopsy analysis | **37** | **22** | **59** |
| Qualitative analysis^1^ | 37 | 20 | 57 |
| Transcriptomic analysis^2^ | 30 | 17 | 47 |
| WGA^3^ | 21 | 16 | 37 |
| Fibrosis | 37 | 20 | 57 |
| PLA^4^ | 34 | 20 | 54 |
| Inflammation^5^ | 36 | 20 | 56 |
| Serum biomarker | **45** | **33** | **78** |
|  |  |  |  |

^1^ Blinded analysis by a cardiovascular pathologist led to the exclusion of 3 biopsies for the subsequent analyses, as non-representative.

^2^ Exclusion due to low RNA quality or low gene number after RNA seq

^3^ Low cardiomyocyte number or not in the good axis for measurement of cell diameter

^4^ Technical issue

^5^ Absence of labeling on one biopsy

**Supplemental Table 2: List of RIN and DV200 for each patient included in the**

| **Sample Codification** | **Condition** | **Bioanalyzer RIN** | **DV200** |
| --- | --- | --- | --- |
| 13EM04 | Non-diabetic patient | 7.7 | 70% |
| 14EM01 | Non-diabetic patient | 6.5 | 71% |
| 15EM01 | Non-diabetic patient | 8.1 | 72% |
| 15EM03 | Non-diabetic patient | 4.4 | 37% |
| 15EM05 | Non-diabetic patient | 6.9 | 70% |
| 15EM10 | Non-diabetic patient | 3.3 | 62% |
| 15EM09 | Non-diabetic patient | 6.7 | 69% |
| 15EM017 | Non-diabetic patient | 4 | 75% |
| 16EM01 | Non-diabetic patient | 7.2 | 71% |
| 16EM03 | Non-diabetic patient | 5.6 | 70% |
| 16EM04 | Non-diabetic patient | 5.8 | 78% |
| 15EM016 | Non-diabetic patient | 6.9 | 70% |
| 16EM06 | Non-diabetic patient | 6.9 | 71% |
| 16EM07 | Non-diabetic patient | 6.3 | 65% |
| 16EM08 | Non-diabetic patient | 6.8 | 71% |
| 17EM03 | Non-diabetic patient | 7.8 | 68% |
| 17EM02 | Non-diabetic patient | 7.1 | 66% |
| 17EM04 | Non-diabetic patient | 7.4 | 73% |
| 17EM05 | Non-diabetic patient | 5.8 | 64% |
| 17EM06 | Non-diabetic patient | 3.5 | 31% |
| 17EM09 | Non-diabetic patient | 7.5 | 69% |
| 18EM02 | Non-diabetic patient | 7.1 | 68% |
| 18EM07 | Non-diabetic patient | 8.3 | 71% |
| 18EM08 | Non-diabetic patient | 6.5 | 69% |
| 18EM10 | Non-diabetic patient | 7.8 | 71% |
| 19EM01 | Non-diabetic patient | 7.4 | 68% |
| 17N12304 | Non-diabetic patient | 7.2 | 70% |
| 17N08114 | Non-diabetic patient | 6.7 | 68% |
| 17N06038 | Non-diabetic patient | 7.5 | 70% |
| 17N05807 | Non-diabetic patient | 7.4 | 71% |
| 13EM05 | Type 2 diabetic patient | 5.8 | 66% |
| 14EM02 | Type 2 diabetic patient | 2.4 | 38% |
| 15EM04 | Type 2 diabetic patient | 4.2 | 62% |
| 15EM06 | Type 2 diabetic patient | 6.4 | 69% |
| 15EM08 | Type 2 diabetic patient | 6.2 | 67% |
| 15EM07 | Type 2 diabetic patient | 6.5 | 69% |
| 16EM02 | Type 2 diabetic patient | 5.6 | 68% |
| 16EM05 | Type 2 diabetic patient | 6.6 | 69% |
| 17EM01 | Type 2 diabetic patient | 7.2 | 69% |
| 17EM07 | Type 2 diabetic patient | 6.9 | 68% |
| 18EM03 | Type 2 diabetic patient | 7 | 74% |
| 19EM02 | Type 2 diabetic patient | 5.9 | 65% |
| 19EM04 | Type 2 diabetic patient | 7.8 | 69% |
| 19EM06 | Type 2 diabetic patient | 7.3 | 69% |
| 17S00077 | Type 2 diabetic patient | 7 | 70% |
| 19S00040 | Type 2 diabetic patient | 7.1 | 62% |
| 17N05428 | Type 2 diabetic patient | 7.4 | 70% |

**Supplemental Table 3**: **Biology parameters**

| **Variable** | **Non-diabetic** | **T2D** | **P Value** |
| --- | --- | --- | --- |
| HbA1c (%) | 5.6 [5.3 ; 5.8] | 6.8 [6.1 ; 7.8]* | ***<0.001*** |
| CrCl (mL/min/1.73^2^) | 80 [71 ; 90] | 86 [70 ; 98] | ns |
| BNP (ng/L) | 53 [38 ; 122] | 97 [54 ; 260] | ns |
| Microalbuminuria (mg/24h) | 9.6 [4.5; 14.8] | 34 [11.9 ; 70.6]* | ***<0.05*** |
| TG (mmol/L) | 1.2 [1 ; 1.7] | 1.5 [1 ; 2.1] | ns |
| Cholesterol total (mmol/L) | 5.7 [4.9 ; 6.1] | 4.0 [3.5 ; 4.5]* | ***<0.001*** |
| HDL (mmol/L) | 1.4 [1.3 ; 1.6] | 1.2 [1 ; 1.4]* | ***<0.01*** |
| LDL (mmol/L) | 3.5 [3.0 ; 4.0] | 2.0 [1.4 ; 2.6]* | ***<0.001*** |

Data are presented as n (%) or median [25^th^ ; 75^th^ percentile]. P value displayed for Fisher exact test was used for categorical variables and the Wilcoxon test was used for continuous variables.

HbA1c, glycated hemoglobin; BNP, brain natriuretic peptide; LDL, low density lipoproteins; TG, triglycerides; HDL, high-density lipoproteins. ns: non-significant.

**Supplemental Table 4**: **List of differentially expressed genes between T2D and non-diabetic patients**

See excel file attached

**Supplemental Figure 1: Flowchart describing all the steps and criteria used for the RNAseq analysis.**


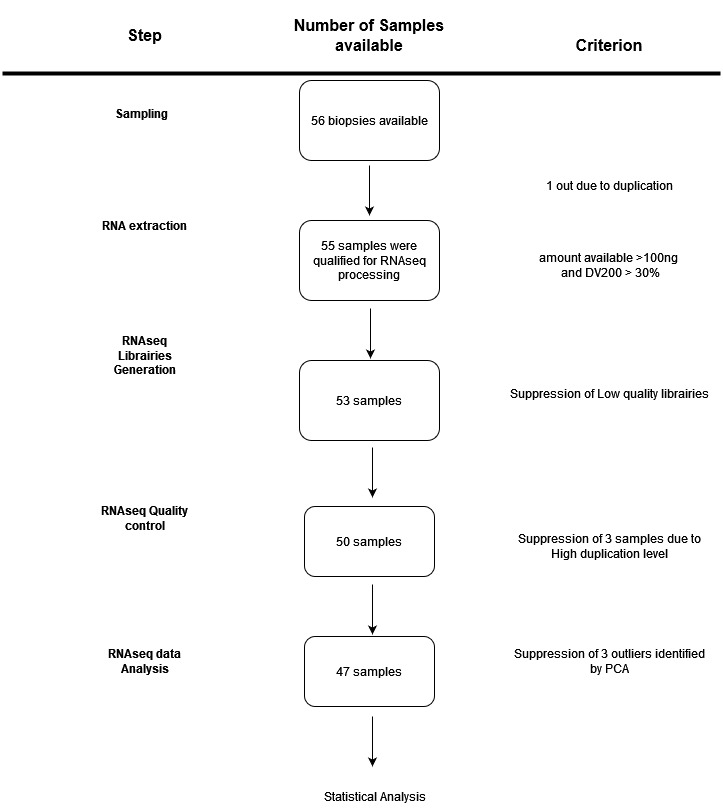


**Supplemental Figure 2**: **Inflammatory and fibrotic circulating markers are not altered by T2D in AS patients.** Quantification of several serum biomarkers of fibrosis (A) and inflammation (B). Statistical analyses: Wilcoxon test. p value signification: ns, not significant; * p<0.05; ** p<0.01; *** p<0.001; **** p<0.0001.

**Supplemental Figure 3**: **Qualitative analysis of hypertrophy, inflammation and fibrosis in LV biopsies from patients with severe AS in the presence or absence of T2D.**

**A)** Representative image of H&E staining of myocardial biopsy. **B)** Qualitative analysis of cellular hypertrophy based on a scoring: qualitative evaluation of the myocyte diameter relative to the scale bar. **C)** Qualitative analysis of inflammation by scoring of the presence of immune infiltrate. **D)** Qualitative analysis of fibrosis based on the following scoring: Sc0= absent; Sc1= interstitial fibrosis but non-mutilating; Sc2= interstitial and mutilating fibrosis in small blocks; Sc3 = interstitial and mutilating fibrosis in large blocks. **E)** Representative image of Sirius Red-stained LV biopsy. **F)** Quantitative analysis of local fibrosis expressed as a percentage of the total area.

Statistical analyses: Fisher and Wilcoxon test. ns: not significant.
